# Targeted Sequencing Analysis of the Leptin Receptor Gene Identifies Variants Associated with Obstructive Sleep Apnoea in Chinese Han Population

**DOI:** 10.1007/s00408-019-00254-z

**Published:** 2019-08-01

**Authors:** Juan Li, Song Yang, Xiaolu Jiao, Yunyun Yang, Haili Sun, Ming Zhang, Yunxiao Yang, Yanwen Qin, Yongxiang Wei

**Affiliations:** 1 Key Laboratory of Upper Airway Dysfunction-Related Cardiovascular Diseases, Beijing An Zhen Hospital, Capital Medical University, Beijing Institute of Heart, Lung, and Blood Vessel Diseases, No. 2 Anzhen Road, Chaoyang District, Beijing, 100029 China; 2 Key Laboratory of Remodeling-Related Cardiovascular Diseases, Institute of Heart, Lung and Blood Vessel Diseases, Beijing An Zhen Hospital, Capital Medical University, Beijing, 100029 China; 3 Otolaryngological Department of Beijing An Zhen Hospital, Capital Medical University, Beijing, 100029 China; 4 Department of Cardiology, Beijing An Zhen Hospital, Capital Medical University, Beijing, 100029 China

**Keywords:** Obstructive sleep apnea, Leptin receptor, Single-nucleotide polymorphisms, Targeted sequencing

## Abstract

**Purpose:**

Obstructive sleep apnea (OSA) is a common sleep disorder that is influenced by various environmental and genetic factors. The potential associations of leptin and leptin receptor (*LEPR*) polymorphisms with OSA have been studied in different populations; however, the results remain inconclusive. The aim of this study was to examine the association between *LEPR* gene polymorphisms and OSA risk.

**Methods:**

A total of 322 samples were used, including 226 OSA subjects and 96 controls. Targeted sequencing of the entire *LEPR* gene was performed in all subjects. Polysomnography was used to diagnose obstructive sleep apnea. The associations between variants and OSA were determined by multivariate regression analyses.

**Results:**

Four single-nucleotide polymorphisms of *LEPR* were identified in all subjects. The genotype frequency of locus rs3790435 was significantly different between the OSA and control groups. Specifically, the variant genotype rs3790435 CC in *LEPR* was associated with a lower risk of OSA (OR 0.462, 95% CI 0.250–0.854, *p* = 0.014) in a recessive model after controlling for potential confounders. After BMI stratification, obese patients with this variant genotype were found to have a lower risk of developing OSA. Moreover, subjects with the rs3790435 CC genotype were found to have a statistically lower apnea–hypopnea index (AHI) and higher nadir oxygen saturation than the TT/CC genotypes without differences in plasma leptin levels.

**Conclusions:**

Our study identified a novel variant of *LEPR* in patients with OSA, and specifically found an association between rs3790435 polymorphisms and OSA risk in Chinese Han subjects.

**Electronic supplementary material:**

The online version of this article (10.1007/s00408-019-00254-z) contains supplementary material, which is available to authorized users.

## Introduction

Obstructive sleep apnea (OSA) is a sleep disorder characterized by recurrent upper airway obstructions leading to sleep fragmentation, daytime sleepiness, fluctuations in blood oxygen levels, and repeated episodes of chronic intermittent hypoxia [1]. OSA affects up to 34% of men and 17% of women, increasing in prevalence with age [2]. The pathogenesis of OSA is thought to involve alterations in the normal neuromuscular activity [3, 4], leading to impaired upper airway patency during sleep. Obesity is a risk factor for OSA, and approximately two thirds of OSA patients are obese [5]; a body mass index (BMI) > 29 kg/m^2^ confers a tenfold increased risk [6]. The development of OSA is complex, and both genetic and environmental factors have been linked to the development of OSA [7]. Genes are thought to play a role via four primary intermediate pathogenic pathways that affect OSA susceptibility: obesity, craniofacial and upper airway morphology, control of ventilation, and control of sleep and circadian rhythm [8].

Leptin is a peptide hormone produced mainly in adipose tissue that regulates energy balance, body weight, metabolism, and endocrine function [9]. Leptin concentrations in blood correlate with body weight and BMI [10]. Studies using animal models have shown that decreased leptin levels or defects in leptin receptors, which prevent leptin from acting on target cells, affect regulation of sleep architecture [11–13], upper airway patency [14, 15], ventilatory function [15–17], and hypercapnic ventilatory response [18]. These findings suggest that leptin may be important to the pathogenesis of OSA through regulation of upper airway patency and diaphragmatic control [19]. Indeed, circulating leptin was significantly increased in OSA patients compared with control subjects matched for age and BMI [20]. Studies like these suggest that leptin might be a biochemical link between sleep disorders and impaired physiological functions.

Leptin acts through the leptin receptor, a single transmembrane domain receptor in the cytokine receptor family [21]. Several epidemiological studies have examined the association between *LEPR* gene polymorphisms and the risk of OSA [22–24]. A recent systematic review and meta-analysis on the role of leptin and *LEPR* polymorphisms in OSA patients found no associations between the risk of OSA and leptin and *LEPR* polymorphisms [25]. However, studies of *LEPR* gene polymorphisms like these are focused mainly on specific sites, such as Gln223Arg, Lys109Arg, and Lys656Asn. They are therefore unable to exclude other single-nucleotide polymorphisms (SNPs) of *LEPR* that may be associated with the risk of OSA. Therefore, the association between leptin receptor gene polymorphisms and susceptibility to OSA remains poorly understood.

Targeted sequencing is a research strategy for enrichment sequencing of genomic regions of interest. The main advantage is that specific regions can be sequenced, which effectively reduces the cost of sequencing, increases the depth of sequencing, and enables more cost-effective study of genetic variation in specific regions. This technology has outstanding advantages in discovering new pathogenic genes or new pathogenic sites on known pathogenic genes, and it can also be applied to areas such as genetic susceptibility research, gene action modification research, and the effects of gene mutation on prognosis [26].

To estimate the overall relationship between OSA and *LEPR* gene polymorphisms, we conducted targeted sequencing of the *LEPR* gene in unrelated Chinese Han subjects with and without OSA to understand the distribution of SNPs and identify new genetic variants in OSA. We also measured the circulating leptin level in OSA patients to determine whether leptin expression in plasma was correlated with variants in the *LEPR* gene.

## Materials and Methods

### Subjects

We conducted a cross-sectional study. Consecutive patients with suspected OSA in the Otolaryngological Department at Beijing An Zhen Hospital from June 2017 to November 2017 were considered potential recruits for this study. All participants received overnight polysomnography (PSG), and OSA patients and normal controls were confirmed based on the American Academy of Sleep Medicine (AASM) Guidelines. Subjects with the following characteristics were excluded from the study: sleep disorders other than OSA (such as upper airway resistance syndrome, restless leg syndrome, narcolepsy), congestive heart failure, pregnancy, cancer, acute infectious diseases, hepatic dysfunction, and abnormal renal function. All participants underwent complete physical examinations, medical interviews, and assessments of demographic, biochemical, and basic clinical variables. Individuals with incomplete information were also excluded. The study flow chart is shown in Supplementary Fig. 1. This study was approved by the Medicine Ethics Committee of Beijing An Zhen Hospital (2,017,005) and was registered in the Chinese Clinical Trial Register (ChiCTR-ROC-17011027). Informed consent was obtained from all participants.

## Polysomnography

The sleep study was conducted using a level II portable diagnostic device (SOMNOscreen; SOMNOmedics GmbH, Randersacker, Germany) approved by the US Food and Drug Administration, including nasal pressure sensors, thermistors, thoracoabdominal belts, blood oxygen probes, and snoring sensors to monitor the breathing. Ventilatory flow at the nose and mouth was measured with thermistors. Ventilatory movements of the chest and abdomen were monitored by inductive plethysmography bands. The arterial oxygen saturation (SpO_2_) was measured transcutaneously with fingertip pulse oximetry. We applied the scoring guidelines and sleep apnea definition of the American Academy of Sleep Medicine [27]. A respiratory event was scored in adults as an apnea if complete cessation of airflow occurred for ≥10 s. Hypopnea was defined as reduced respiratory airflow by 30% with a 4% decrease in oxygen saturation. Apnea events were classified as obstructive, mixed, or central, according to the presence or absence of breathing efforts with thoracoabdominal paradox. The apnea–hypopnea index (AHI) was determined by dividing the number of the total apnea/hypopnea events by the estimated hours of sleep [28]. A diagnosis of OSA was defined as AHI ≥ 5 events per hour, according to the American Academy of Sleep Medicine guidelines [28, 29]. OSA severity was quantified via the AHI: mild OSA (AHI: 5–14.9), moderate OSA (AHI: 15–29.9), or severe OSA (AHI ≥ 30) [30]. Subjects with AHI < 5 served as controls. Finally, a total of 322 subjects were included in this study, including 226 OSA patients and 96 controls.

## Data Collection

Subjects were classified as non-smokers if they had never smoked or had stopped smoking ≥ 1 year before enrollment in the study; all other subjects were classified as smokers. Subjects were diagnosed with hypertension if they had a systolic blood pressure of > 140 mmHg or diastolic blood pressure of > 90 mmHg [11]. Subjects were defined as drinkers if daily alcohol intake exceeded ≥ 3 times a week. Obesity was defined as BMI ≥ 28 kg/m^2^, according to the recommendations of the Health Promotion Administration [31]. High-density lipoprotein cholesterol levels (HDL-C), low-density lipoprotein cholesterol levels (LDL-C), total cholesterol levels (TC), triglyceride levels (TG), and fasting blood glucose (FBG) levels were measured according to standard laboratory methods at Beijing An Zhen Hospital. Circulating leptin levels were determined by enzyme-linked immunosorbent assay.

## DNA Template Preparation and Amplification

Venous blood samples were collected after the participants had fasted overnight. Blood samples were centrifuged at 2943×*g* at 4 °C for 10 min. Serum and whole blood samples were then stored at – 80 °C prior to analysis. Genomic DNA was extracted from 200 μl of blood according to the protocol provided in the QIAamp DNA Mini Kit (Qiagen, Hilden, Germany). DNA concentration was determined using NanoDrop 2000c spectrophotometer (Thermo Fisher Scientific Inc, Waltham, MA). A multiplex PCR amplification strategy was designed online (Ion Ampliseq® Designer; https://www.ampliseq.com) to amplify the target region (for primer sequences, see Supplementary Table 1), and the detailed design process is presented at https://www.ampliseq.com/help/pipelineDetails.action. Primers were designed to provide maximum coverage, and the ordered amplicon covered approximately 100% of the target sequence (Supplementary Table 2). Details are described in Supplemental Appendix 1.

## Targeted Sequencing

*LEPR* genes were sequenced via Ion Torrent semiconductor sequencing (Life Technologies, Carlsbad, CA, USA). Enriched Ion Sphere Particles carrying numerous copies of the same DNA fragments were subjected to sequencing on an Ion 318 Chip to sequence pooled libraries with 64 samples. Sequencing was performed using the sequencing kit (Ion PGM Sequencing Kit; Life Technologies) in accordance with the manufacturer’s instructions with the 400-bp single end-run configuration. Computational analysis is outlined in Supplemental Appendix 2.

### Statistical Analysis

Continuous variables are expressed as a mean ± standard deviation or median (interquartile range), and categorical variables are expressed as a numeral (percentage). Independent Student’s *t* tests for normal distribution and Kruskal–Wallis H tests were used to compare the differences between non-normal continuous variables among genotypes under three genetic models. Chi-squared tests and Fisher’s exact tests were used to analyze categorical variables. Deviations of genotype frequencies from the Hardy–Weinberg assumption were assessed using chi-squared tests. The associations between OSA and variants were determined by logistic regression analyses. Mann–Whitney U test or Kruskal–Wallis H test was used to compare the difference between AHI, nadir SpO_2_, and leptin levels among genotypes under three genetic models. The three genotypes of the marker were denoted as aa, Aa, and AA, and these were coded as (0, 1, 2) (0, 1, 1) and (0, 0, 1) under additive, dominant, and recessive models, respectively. A is assumed to be the risk allele. All probability values were two-sided and a *p* value < 0.05 was considered statistically significant. All analyses were performed with R (https://www.R-project.org) and EmpowerStats software (www.empowerstats.com, X&Y solutions, Inc., Boston, MA).

## Results

### Baseline Characteristics of Participants

The present study included 226 OSA patients and 96 controls. The clinical characteristics of these individuals are presented in Table 1. There were no differences in mean age (*p* = 0.067), systolic blood pressure (*p* = 0.346), diastolic blood pressure (*p* = 0.058), HDL-C level (*p* = 0.053), LDL-C level (*p* = 0.836), TC level (*p* = 0.725), or FBG level (*p* = 0.133) between the two groups. BMI, TG, and polysomnographic parameters in control subjects were significantly different from the corresponding parameters in patients with OSA; the OSA patients had higher BMI (*p* < 0.001), TG level (*p* = 0.006), AHI (*p* < 0.001), and the lowest oxygen saturation (*p* < 0.001) measurements. There were more male (*p* = 0.002) and smoker (*p* = 0.017) patients in the OSA group compared with control subjects. The leptin levels were not statistically different between OSA and control groups (*p* = 0.564), Table 1.

**Table 1:** Baseline characteristics of participants

Measure	OSA	Control	*p*
Number of subjects	226	96	-
Age (years)	55.3 ± 10.8	52.7 ± 13.8	0.067
Male (n, %)	194 (85.8%)	69 (71.1%)	0.002*
BMI (kg/m2)	27.3 ± 3.6	23.9 ± 3.6	< 0.001**
SBP (mmHg)	125.7 ± 18.9	123.5 ± 18.0	0.346
DBP (mmHg)	77.4 ± 13.1	74.5 ± 11.7	0.058
TC (mmol/L)	4.4 ± 1.1	4.4 ± 1.1	0.725
TG (mmol/L)	1.5 ± 1.1#	1.4 ± 0.6#	0.006*
HDL-C (mmol/L)	1.1 ± 0.3	1.1 ± 0.3	0.053
LDL-C (mmol/L)	2.6 ± 1.0	2.6 ± 0.8	0.836
FPG (mmol/L)	4.5 ± 2.0	4.8 ± 1.6	0.133
AHI (times/h)	24.9 ± 21.0#	2.7 ± 2.0#	< 0.001**
LSaO2 (%)	85.0 ± 7.0#	89.5 ± 4.0#	< 0.001**
Drinker (n, %)	96 (42.5%)	37 (38.1%)	0.468
Smoker (n, %)	107 (47.3%)	32 (33.0%)	0.017*
Leptin (pg/mL)	6176.2 ± 6878.7#	5978.9 ± 9587.0#	0.564

Values are expressed as mean ± standard deviation, median ± interquartile range, or n (%). Differences between groups were analyzed by independent Student * t* test, Fisher’s exact test, * χ*^2^ test, or Wilcoxon test

^#^Data were asymmetrically distributed. *p < 0.05, **p 0.001

*OSA* Obstructive sleep apnea, * BMI* body mass index, * SBP* systolic blood pressure, * DBP* diastolic blood pressure, * FPG* fasting plasma glucose, * TG* triglycerides, * TC* total cholesterol, * LDL-C* low-density lipoprotein cholesterol, * HDL-C* high-density lipoprotein cholesterol, * AHI* apnea–hypopnea index, * LSaO2* lowest oxygen saturation

## Association with OSA

A targeted next-generation sequencing approach was used to analyze the *LEPR* gene in 226 OSA patients and 96 controls.

The customized targeted sequencing panel was generated to capture exons, intron/exon boundaries, and flanking untranslated regions (UTRs) of *LEPR*. A total of 80 nucleotide variants were found in 316 samples, of which 54 were synonymous variants, intron variants, and variants not present in the 1000 Genomes Database, the EXAC database, the dbSNP database, or the ESP6500 database. After excluding deletion/insertion polymorphisms and those variants without rs-number, four SNPs (minimum allele frequency ≥ 0.01) remained. The allele frequency distribution is shown in Supplementary 3. All SNPs were consistent with the Hardy–Weinberg equilibrium.

Of these SNPs, the genotype and allele frequencies at rs3790435 differed significantly (*p* = 0.036 and *p* = 0.02, respectively) between OSA and control groups (Supplementary 4). After adjusting for age, sex, BMI, TG, TC, LDL-C, HDL-C, FBG, smoker designation, and drinker designation, the variant genotype rs3790435 CC in *LEPR* (OR 0.462, 95% CI 0.250–0.854, *p* = 0.014) was found to be negatively associated with a diagnosis of OSA compared with wild-type carriers, according to the recessive model (Table 2). As obesity is common in OSA patients and is also a risk factor for OSA, we conducted further stratified analysis to explore genotype frequency differences in OSA patients with and without obesity. As shown in Table 3, obese subjects with the rs3790435 CC genotype variant had a lower risk of OSA (OR 0.191, 95% CI 0.041–0.878, *p* = 0.033). There were no significant findings for the other three SNPs.

**Table 2:** Multivariate logistic regression analyses of four SNPs in *LEPR* gene with the risk of OSA

SNP	Genotypes	Unadjusted		Model 1		Model 2	
		OR (95% CI)	*p* Value	OR (95% CI)	*p* Value	OR (95% CI)	*p* Value
rs3790435 Additive	TT(*n* = 20)	1		1		1	
	TC (*n* = 94)	1.316 (0.425–4.075)	0.634	1.068 (0.310–3.677)	0.917	1.434 (0.409–5.020)	0.573
	CC (*n* = 208)	0.630 (0.220–1.802)	0.389	0.528 (0.168–1.661)	0.275	0.588 (0.186–1.861)	0.366
Dominant	TT (*n* = 20)	1		1		1	
	TC + CC( *n* = 302)	0.773 (0.273–2.190)	0.628	0.638 (0.205–1.991)	0.439	0.747 (0.239–2.337)	0.616
Recessive	TT + TC (*n* = 114)	1		1		1	
	CC (*n* = 208)	0.524 (0.311–0.885)	0.016*	0.521 (0.289–0.938)	0.030*	0.462 (0.250–0.854)	0.014*
rs3790431 Additive	AA (*n* = 291)	1		1		1	
	AG (*n* = 29)	1.119 (0.477–2.625)	0.795	1.045 (0.391–2.794)	0.931	1.003 (0.370–2.715)	0.996
	GG (*n* = 2)	0.426 (0.026–6.896)	0.548	0.719 (0.028–18.802)	0.843	0.639 (0.024–16.959)	0.789
Dominant	AA (*n* = 291)	1		1		1	
	AG + GG (*n* = 31)	1.042 (0.461–2.355)	0.920	1.017 (0.393–2.632)	0.972	0.970 (0.371–2.535)	0.950
Recessive	AA + AG (*n* = 320)	1		1		1	
	GG (*n* = 2)	0.422 (0.026–6.821)	0.544	0.714 (0.027–18.610)	0.840	0.639 (0.024–16.902)	0.788
rs13306519 Additive	CC (*n* = 215)	1		1		1	
	CG (*n* = 96)	1.193 (0.698–2.037)	0.519	1.312 (0.719–2.393)	0.377	1.236 (0.670–2.279)	0.497
	GG (*n* = 11)	0.775 (0.219–2.739)	0.692	1.661 (0.383–7.211)	0.498	1.641 (0.349–7.718)	0.530
Dominant	CC (*n* = 215)	1		1		1	
	CG + GG (*n* = 107)	1.137 (0.681–1.897)	0.623	1.346 (0.754–2.402)	0.315	1.272 (0.704–2.298)	0.425
Recessive	CC + CG (*n* = 311)	1		1		1	
	GG (*n* = 11)	0.735 (0.210–2.572)	0.630	1.522 (0.356–6.497)	0.571	1.531 (0.330–7.099)	0.586
rs3206172 Additive	CC (*n* = 264)	1		1		1	
	CG (*n* = 56)	0.962 (0.513–1.803)	0.904	0.967 (0.481–1.945)	0.925	1.038 (0.507–2.124)	0.919
	GG (*n* = 2)	0.419 (0.026–6.789)	0.541	0.709 (0.027–18.550)	0.836	0.643 (0.024–17.049)	0.792
Dominant	CC (v264)	1		1		1	
	CG + GG (*n* = 58)	0.932 (0.503–1.725)	0.822	0.957 (0.481–1.905)	0.900	1.019 (0.504–2.060)	0.958
Recessive	CC + CG (*n* = 320)	1		1		1	
	GG (*n* = 2)	0.422 (0.026–6.821)	0.544	0.714 (0.027–18.610)	0.840	0.639 (0.024–16.902)	0.788

Model 1: adjusted for age, sex, BMI, Smoker, and Drinker

Model 2: adjusted for Model 1 + TG, TC, LDL-C, HDL-C, and FBG

^*^p < 0.05

**Table 3:** Multivariate association analysis of rs3790435 in *LEPR* gene with the risk of OSA stratified by BMI

Model	Genotypes	No	Non-obese		Obese		
			OR (95% CI)	*p*	No	OR (95% CI)	*p*
Additive	TT	14	1		6	1	
	TC	54	1.223 (0.307–4.875)	0.775	40	1.551 (0.069–35.071)	0.783
	CC	137	0.691 (0.193–2.476)	0.571	71	0.274 (0.015–5.166)	0.388
Dominant	TT	14	1		6	1	
	TC + CC	191	0.796 (0.224–2.821)	0.723	111	0.542 (0.040–7.413)	0.646
Recessive	TT + TC	68	1		46	1	
	CC	137	0.621 (0.323–1.195)	0.154	71	0.191 (0.041–0.878)	0.033*

^*^p < 0.05 adjusted for age, sex, TG, TC, LDL-C, HDL-C, FBG, smoker, and drinker

We then conducted a genotype–phenotype correlation analysis, which showed that subjects with the rs3790435 CC genotype had a significantly lower AHI (median: 17.75 vs. 21.25 events/h, *p* = 0.046) and higher nadir oxygen saturation (median: 87% vs. 86%, *p* = 0.018) compared with subjects with the TT/CC genotype (Table 4). Plasma leptin levels were also measured in OSA patients (Table 5), but no associations between leptin levels and rs3790435 were found in OSA using three genetic models.

**Table 4:** Differences in clinical indicators among *LEPR* SNP genotypes in subjects

SNP	Model	Genotype	AHI		LSaO_2_ (%)	
			Median ± IQR	*p*	Median ± IQR	*p*
rs3790435	Additive	TT (*n* = 20)	17.40 ± 18.0	0.052	87.50 ± 8.0	0.039*
		TC (*n* = 94)	23.15 ± 30.0		86.00 ± 9.0	
		CC (*n* = 208)	17.75 ± 30.0		87.00 ± 9.0	
	Dominant	TT (*n* = 20)	17.40 ± 18.0	0.545	87.50 ± 8.0	0.945
		TC + CC (*n* = 302)	19.50 ± 31.0		87.00 ± 8.0	
	Recessive	TT + TC (*n* = 114)	21.25 ± 28.0	0.046*	86.00 ± 8.0	0.018*
		CC (*n* = 208)	17.75 ± 30.0		87.00 ± 9.0	

Data expressed are Median ± IQR. Mann–Whitney U test was used for two groups’ analysis and Kruskal Wallis H test was used for three groups

*IQR* interquartile range, *AHI* apnea–hypopnea index, *LSaO*_*2*_ lowest oxygen saturation

**Table 5:** Association of *LEPR* gene polymorphism with serum leptin level in the OSA patients

SNP	Model	Genotype	Leptin (pg/mL)	
			Median ± IQR	*p*
rs3790435	Additive	TT (*n* = 15)	5393.25 ± 7685.50	0.691
		TC (*n* = 75)	7148.68 ± 5812.70	
		CC (*n* = 136)	6089.85 ± 7468.40	
	Dominant	TT (*n* = 15)	5393.25 ± 7685.50	0.472
		TC + CC (*n* = 211)	6192.32 ± 6890.82	
	Recessive	TT + TC (*n* = 90)	6551.12 ± 5852.67	0.828
		CC (*n* = 136)	6089.85 ± 7468.40	

Data expressed are Median ± IQR. Mann–Whitney U test was used for two groups’ analysis and Kruskal Wallis H test was used for three groups

*IQR* interquartile range

## Discussion

OSA is heritable, and there is evidence of genetic contributions to OSA susceptibility [32]. Around 40% of the variance in AHI is attributable to genetic factors [33]. In this study, we detected a novel genetic variant of the *LEPR* gene that is significantly associated with OSA. The variant genotype CC in *LEPR* rs3790435 was associated with a lower OSA risk in recessive modeling, especially among obese subjects. Carriers with this variant genotype had lower AHI and higher nadir oxygen saturation measurements.

Leptin is a protein hormone secreted by adipose tissue that participates in energy metabolism and food intake. In recent years, a growing body of research has suggested that leptin may be a biomarker for OSA [34, 35]. The relationship between leptin and OSA may be assessed by examining pertinent risk factors and pathological processes. Leptin is associated with obesity, which is a risk factor for OSA. It also acts as a potent respiratory stimulant, binding the leptin receptor in the carotid bodies to stimulate breathing and the hypoxic ventilatory response; it may therefore protect against sleep-disordered breathing in obesity [36]. Leptin-deficient mice have marked decreases in active pharyngeal neuromuscular responses and a higher frequency of inspiratory flow limitation than wild-type mice, independent of body weight [15, 17]. In mouse studies, leptin-deficient or leptin receptor knockout mice had significantly disrupted sleep architecture with an elevated number of arousals from sleep and increased stage shifts compared with wild-type mice [11, 13]. Our study found that rs3790435 CC variants were associated with a lower risk of OSA in obese subjects, suggesting that *LEPR* might influence the occurrence of OSA with obesity.

Previous genome-wide linkage and association studies have revealed multiple common genes and loci that are linked to OSA, including the leptin receptor. There are several common variants of the *LEPR* genes, and the potential associations of these variants with OSA have been evaluated in different populations with inconsistent results. The polymorphism Gln223Arg in *LEPR* was found to have a significant correlation with OSA. Patients who were carriers of the Arg allele were found to develop OSA more often than carriers of the Gln allele [22, 37]. The *LEPR* K656N gene polymorphism was found to be associated with AHI; subjects with an NN variant genotype had lower AHI measurements than wild-type subjects [38]. Our study also found significantly lower AHI measurements in individuals with the *LEPR* rs3790435 CC genotype compared with those with a TT/CC genotype.

However, there were no associations between three common SNPs (the Lys109Arg, Gln223Arg, and Lys656Asn polymorphisms) and OSA in another study [23]. Consistent with two recent systematic reviews and a meta-analysis [25, 39] on leptin and *LEPR* gene polymorphisms in OSA, no polymorphisms (Gln233Arg, Lys656Asn, Lys109Arg, 19A/G, Pro1019Arg, or 2548G/A) were found to be associated with OSA risk. These results should be interpreted with caution, however, as the number of studies included and sample sizes were relatively small, restricting the power of the meta-analysis. Furthermore, these studies focused mainly on specific predefined SNPs.

To the best of our knowledge, this is the first report of *LEPR* rs3790435 variants associated with OSA in a Chinese Han population. Previous studies have examined associations between preselected SNPs and OSA risk; in contrast, we used targeted sequencing to discover genetic susceptibility loci without prior knowledge of functionality or position within the genome. Care was taken to avoid bias in this study. Male sex, age, and obesity are major risk factors for OSA [40, 41]. Smoking is also commonly linked with OSA [40]. We adjusted for age, sex, BMI, and smoking to avoid confounding effects. Leptin acts as an adipokine that regulates lipid metabolism. To avoid confounding effects, we also adjusted for TG, TC, LDL-C, and HDL-C. Also, genomic DNA was extracted and targeted sequencing was carried out by a trained experimenter who was unaware of the patients’ clinical data. During statistical analysis, adjustments were made for the confounding risk factors for OSA and *LEPR*. Finally, subjects in this study were consecutively recruited to reduce the effects of outcome-selection bias.

Some limitations of this study should be considered. First, the sample size is relatively small, and therefore, the statistical power may not be high enough to definitively explore associations. Second, this was a cross-sectional study with limited power; prospective cohort studies are needed to confirm the variants in our study. Finally, the role of the *LEPR* polymorphism in OSA pathogenesis remains unclear, and requires further functional studies.

In conclusion, we identified a novel variant of *LEPR* in patients with OSA, and predicted that this variant is associated with OSA risk. In this study, people with the *LEPR* rs3790435 CC genotype have a decreased risk of developing OSA, a lower AHI, and a higher nadir oxygen saturation compared with those with the TT/CC genotype after adjusting for confounding variables. Still, these findings warrant further investigation and validation with larger patient populations, leading to a better, more comprehensive understanding of the association between *LEPR* polymorphisms and OSA risk.

## Electronic supplementary material

Below is the link to the electronic supplementary material.

Electronic supplementary material 1 (DOCX 95 kb)
