# Supplementary material for: Targeted Sequencing Analysis of the Leptin Receptor Gene Identifies Variants Associated with Obstructive Sleep Apnoea in Chinese Han Population
Source: Lung. 2019 Aug 1;197(5):577–84. doi: 10.1007/s00408-019-00254-z (PMC6778532; doi:10.1007/s00408-019-00254-z)
Supplement: Supplementary file 1 — Electronic supplementary material 1 (DOCX 95 kb) [file 408_2019_254_MOESM1_ESM.docx]

**Supplementary Figure 1**


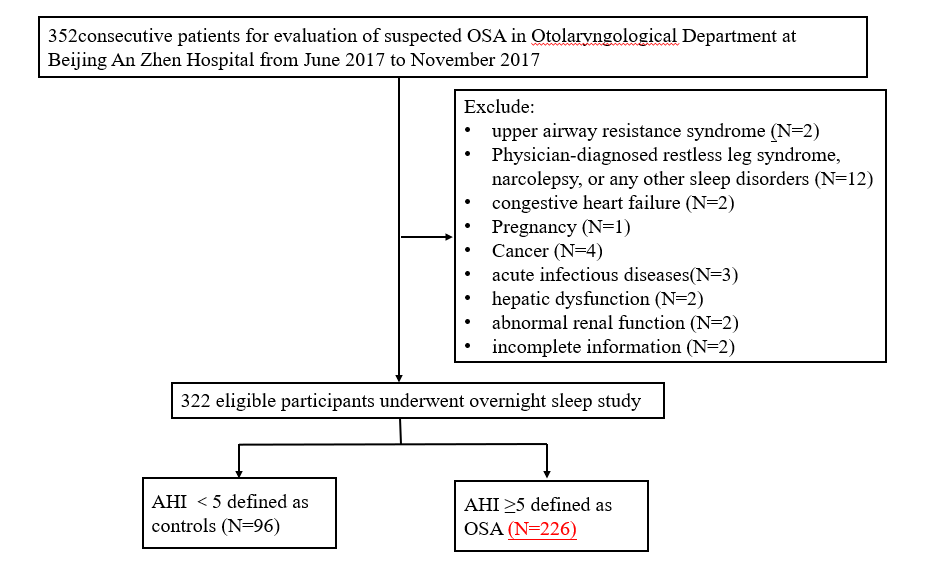


**Supplemental Appendix 1**

A total of 10 ng of DNA per sample was used for enrichment by multiplex PCR, and each DNA pool was amplified with the Ion Ampliseq™ Library Kit in conjunction with the Ion Ampliseq™ “custom Primer Pool”; the manufacturer’s protocols were followed (Life Technologies, Darmstadt, Germany).

After each pool had undergone 17 PCR cycles, the PCR primers were removed with FuPa Reagent (Life Technologies) and the amplicons were ligated to the sequencing adaptors with short stretches of barcodes that enabled sample multiplexing for subsequent steps (Ion Xpress™ Barcode Adapters Kit; Life Technologies). After purification with AMPure XP beads (Beckman Coulter, Krefeld, Germany), the barcoded libraries were quantified with a Qubit® 2.0 Fluorimeter (Life Technologies) and normalized for the DNA concentration to a final concentration of 20 pmol/L using the Ion Library Equalizer™ Kit (Life Technologies). Equalized barcoded libraries from 64 samples at a time were pooled. To clonally amplify the library DNA onto the Ion Sphere Particles (Life Technologies), the library pool was subjected to emulsion PCR using an IT OneTouch template kit on an IT OneTouch system (Life Technologies) following the manufacturer’s protocol.

**Supplemental Appendix 2**

Torrent Suite Software (Version 4.4.2; Life Technologies, Darmstadt, Germany) was used to generate read alignments from the raw data (unmapped BAM-files). The read alignments were filtered by the software into mapped BAM-files using the reference genomic sequence (hg19) of the target genes. Variant calling was performed with the Torrent Variant Caller Plugin (minimum allele frequency, 0.2; minimum quality, 20; minimum coverage, 20; and minimum coverage on either strand, 3). Annotation of variants was performed using Ion Reporter Software (Version 4.4; Life Technologies, Darmstadt, Germany) for the Variant Call Format files. The annotation included genomic and complementary DNA positions, genetic reference sequences, amino acid changes, and related information available from public databases, such as the 1000 Genomes Project, Single Nucleotide Polymorphism Database (dbSNP147) (National Center for Biotechnology Information, http://www.ncbi.nlm.nih.gov/SNP/), Exome Aggregation Consortium (ExAC03) (http://exac.broadinstitute.org), ClinVar, Online Mendelian Inheritance in Man (OMIM), and Human Gene Mutation Database (HGMD).

**Supplementary Table 1** Primers used for *LEPR*

| Ion_AmpliSeq_Fwd_Primer* | Ion_AmpliSeq_Rev_Primer* | Name | Amplicon_Start | Insert_Start | Insert_Stop | Amplicon_Stop |
| --- | --- | --- | --- | --- | --- | --- |
| CCAATTACTCCTTGGAGATTTAAGTTGTCT | AGCCATAAGACATCTATTTCATACAGGTAT | LEPR | 66036194 | 66036224 | 66036537 | 66036567 |
| CTATTTCTGAAGGCAGAGAACACAGA | AGAAATACTTTTCAGCCATATTTGAAGCAC | LEPR | 66075489 | 66075515 | 66075833 | 66075863 |
| GTCCTTGGATAAAGTCACCTTTTAAGTACT | ATTTAGTATGCAGAGGGTAATTGCTATGG | LEPR | 66061997 | 66062027 | 66062337 | 66062366 |
| GCCTCTGTGTTTAGAAGATAGTAAAGACAT | AATCTCTTTTGAGGGAACAATCTTGTTTTC | LEPR | 66066830 | 66066860 | 66067148 | 66067178 |
| AACTTGAATTTTCATTATGGACCCTCCTTA | ACTTTCCTCTTTCAGGAAGCAAATAATC | LEPR | 66067401 | 66067431 | 66067729 | 66067757 |
| TAATCCCTTTCCTTTTATGTTTTCCACAGA | CACTGATACACCTATACTATGGTTTGCATT | LEPR | 66031137 | 66031167 | 66031425 | 66031455 |
| CAGGTACTACTACTAGCTTTCAGAGTAACA | GGCTTTTCCCAAGATATTTTCAATAATCCA | LEPR | 66074176 | 66074206 | 66074484 | 66074514 |
| TCATCATTATTTTGAAAGCTGTTTTCGCT | AACCATCTGAAAACTGCCTTTTTGG | LEPR | 66058045 | 66058074 | 66058372 | 66058397 |
| ACCCTACGGAGGCATAGTTGAT | CCTGCATCCTGCTATTCTTGAAAGTATATA | LEPR | 66088470 | 66088492 | 66088798 | 66088828 |
| GCTTAAGTTTGTTTTGAAACTCCCTTGA | CTTTGGACTCTTCCATGATTCTATCAGAA | LEPR | 66085445 | 66085473 | 66085790 | 66085819 |
| AGCTCAGTTAGTATAAAAAGCACTGCA | TTGCAGGCTGCTTGAAAGATAATTTAAAAT | LEPR | 66081629 | 66081656 | 66081972 | 66082002 |
| GTGTTGCTTCTGAGCAGTCAGA | AAAAAGAACCACTCCAGATTCATCACT | LEPR | 66095809 | 66095831 | 66096149 | 66096176 |
| ATCTAAACAGAGAACGGACATTCTTTGAA | GTAATGACAGCAACAGCCATAATTTTCTT | LEPR | 66098825 | 66098854 | 66099158 | 66099187 |
| GTTCTTTTGAATATCTCCTGGCATTTTTGT | TCACCATGAACTATTCATAAAAGCTCCAA | LEPR | 66099395 | 66099425 | 66099732 | 66099761 |
| AAAGTAGAAAAGAACTCTACAAAAACGGGA | ATGTCATACTCAGTAACTGTTGAAAGCA | LEPR | 66099956 | 66099986 | 66100289 | 66100317 |
| ACAGTTCAGTGCATATATAGTAGAAGCCT | TGTTCTTCATAATGGCAAGCATATTCCATA | LEPR | 66100468 | 66100497 | 66100785 | 66100815 |
| AATTTTTCAAGGTGATGTATCAACAGCTTT | GGACATGTGAAATGAAGACAAATACACAAG | LEPR | 66100946 | 66100976 | 66101116 | 66101146 |
| CAACTGTGGTCTCTCTACTTTCAACA | TCAAAAGTTCATCCAATCCTTCTGAGAAT | LEPR | 66102010 | 66102036 | 66102355 | 66102384 |
| AGTAAGAAGACTTTTGCATCTTACATGC | CTAGCTGGAAACAAGGGACCTT | LEPR | 66102600 | 66102628 | 66102931 | 66102953 |
| TCCAGAAAATTTACTCATCCGCCAAA | CCCACAAAGGGACTGTGTTCATAA | LEPR | 65890857 | 65890883 | 65891183 | 65891207 |
| TTCCTCAAGTTTCTGAGTTGTGTAAATTG | GTAAGGACTTTTGCCGTAAAGATTCTT | LEPR | 66086993 | 66087022 | 66087158 | 66087185 |
| AGTTGTCATGGATATAAAAGGTCTGCA | GCCACTGTACATCTTAGCTCTCTTTAAATA | LEPR | 66075770 | 66075797 | 66076110 | 66076140 |
| GTGGCCAGATAACTACTGTGTAAGATATT | CTCCTTTCATTTTAAGGCCTCAAAATGT | LEPR | 66064218 | 66064247 | 66064564 | 66064592 |
| TTCTTTTTCTCTCAGAATAGGCAATGGA | GGAAATGTAGGGATGCAAGAGGT | LEPR | 66037861 | 66037889 | 66038212 | 66038235 |
| TGGGTCTAATGTTTCTTTTCACTGCA | GAGGGTCCATAATGAAAATTCAAGTTGTG | LEPR | 66067112 | 66067138 | 66067397 | 66067426 |
| TCTGAAATCTTACCTATGGACCACCAT | ACAAATAAGAACATTCACACCCACTGTAT | LEPR | 66091680 | 66091707 | 66092018 | 66092047 |
| TGTCAGCTTAAAGAGAAAACATGAAGTGT | GCAAAAGGAAGTAGTCATAGGTTGAATTT | LEPR | 66035890 | 66035919 | 66036234 | 66036263 |
| TCCATCCAGTGTGAAAGCAGAAATT | GCATTTAATGAGGAAAGCTGAAGTTCTAAG | LEPR | 66074449 | 66074474 | 66074594 | 66074624 |
| CAATATAGGCCTGAAGTGTTAGAAGATTCA | TGTTAAGCAAAGTGAGATAAGCTAGCAAAT | LEPR | 66058332 | 66058362 | 66058625 | 66058655 |
| GGGACTGCTGTTTTAAACAACAAATCA | TGCATCAATCTGCATACAAATCTGC | LEPR | 66070625 | 66070652 | 66070955 | 66070980 |
| CCTCCTCCTCTCCTGAGAGTTG | CCCATTCTCGAGTTACCCAGA | LEPR | 65991344 | 65991366 | 65991550 | 65991571 |
| CCGCGTTTGCGAGCTAAG | ACGAAGGGAGCGGTCGATC | LEPR | 65886246 | 65886264 | 65886558 | 65886577 |
| GGTTCTCCCTCTTTAAATTTTTCCAGC | GCACATTGGGTTCATCTGTAGTGAT | LEPR | 66098515 | 66098542 | 66098864 | 66098889 |
| TGGCTTTTGATTTGTCATATTCCTGGT | CAAGGATATTGAAAACTGAAATTCCCAGAT | LEPR | 66099121 | 66099148 | 66099439 | 66099469 |
| GGAAATGGGCAGTGATGCAAAAA | ACACCTATTAGAAGTCAAGAAGGGAAGAA | LEPR | 66099699 | 66099722 | 66099996 | 66100025 |
| ATATTCTATCTGAATGCATTGGAAGGCAT | AGAGAAAACTGTTCCTATGCCTGTTTTT | LEPR | 66100251 | 66100280 | 66100566 | 66100594 |
| ATTTTGCGCTTGGCATATTTATTCCT | GGCAAAAAGCTCAATTCATACAATGTAAAC | LEPR | 66100693 | 66100719 | 66101003 | 66101033 |
| AACGGGCTCTTCCTTATGCTTT | CTGGTCACTAATACAAACAGAACCCTTT | LEPR | 66101700 | 66101722 | 66102046 | 66102074 |
| TTATCAGATCAGCATCCCAACATAATTTCA | CATGATCTTATGAGTCTGAGTAGAACAAGT | LEPR | 66102315 | 66102345 | 66102638 | 66102668 |
| GTAGACACGCTCTTCTATTTTATTCCCAA | GGCTCATTAGTCTTCACAAGTATAACCC | LEPR | 66102892 | 66102921 | 66103190 | 66103218 |
| TCTGTCTTCTCTTCCTTATTCCCTTTAGT | TCTGTAGTGTCATATTAATCCATGAGAGCA | LEPR | 66083538 | 66083567 | 66083854 | 66083884 |

**Supplementary Table 2** AmpliSeq™ amplicons and coverage details of obstructive sleep apnea-targeted sequencing assay

| Target_ID | Gene_Symbol | Chromosome | Chr_Start | Chr_End | Num_Amplicons | Total_Bases | Covered_Bases | Missed_Bases | Coverage |
| --- | --- | --- | --- | --- | --- | --- | --- | --- | --- |
| 516357 | LEPR | chr1 | 65886329 | 65886428 | 1 | 99 | 99 | 0 | 1 |
| 516358 | LEPR | chr1 | 65890980 | 65891066 | 1 | 86 | 86 | 0 | 1 |
| 516360 | LEPR | chr1 | 65991366 | 65991506 | 1 | 140 | 140 | 0 | 1 |
| 516359 | LEPR | chr1 | 66031223 | 66031293 | 1 | 70 | 70 | 0 | 1 |
| 502956 | LEPR | chr1 | 66036150 | 66036490 | 2 | 340 | 340 | 0 | 1 |
| 502951 | LEPR | chr1 | 66038003 | 66038137 | 1 | 134 | 134 | 0 | 1 |
| 502960 | LEPR | chr1 | 66058334 | 66058553 | 2 | 219 | 219 | 0 | 1 |
| 502949 | LEPR | chr1 | 66062125 | 66062281 | 1 | 156 | 156 | 0 | 1 |
| 502947 | LEPR | chr1 | 66064337 | 66064492 | 1 | 155 | 155 | 0 | 1 |
| 502953 | LEPR | chr1 | 66067069 | 66067370 | 2 | 301 | 301 | 0 | 1 |
| 502955 | LEPR | chr1 | 66067520 | 66067648 | 1 | 128 | 128 | 0 | 1 |
| 502965 | LEPR | chr1 | 66070715 | 66070925 | 1 | 210 | 210 | 0 | 1 |
| 502959 | LEPR | chr1 | 66074430 | 66074589 | 2 | 159 | 159 | 0 | 1 |
| 502948 | LEPR | chr1 | 66075624 | 66075794 | 1 | 170 | 170 | 0 | 1 |
| 502946 | LEPR | chr1 | 66075891 | 66075984 | 1 | 93 | 93 | 0 | 1 |
| 502966 | LEPR | chr1 | 66081685 | 66081912 | 1 | 227 | 227 | 0 | 1 |
| 502962 | LEPR | chr1 | 66083641 | 66083834 | 1 | 193 | 193 | 0 | 1 |
| 502963 | LEPR | chr1 | 66085605 | 66085711 | 1 | 106 | 106 | 0 | 1 |
| 502952 | LEPR | chr1 | 66087030 | 66087146 | 1 | 116 | 116 | 0 | 1 |
| 502961 | LEPR | chr1 | 66088583 | 66088669 | 1 | 86 | 86 | 0 | 1 |
| 516364 | LEPR | chr1 | 66091800 | 66091922 | 1 | 122 | 122 | 0 | 1 |
| 516361 | LEPR | chr1 | 66095879 | 66096100 | 1 | 221 | 221 | 0 | 1 |
| 516362 | LEPR | chr1 | 66098829 | 66101116 | 10 | 2287 | 2287 | 0 | 1 |
| 516363 | LEPR | chr1 | 66101868 | 66103181 | 5 | 1313 | 1313 | 0 | 1 |

**Supplementary Table 3** Hardy-Weinberg equilibrium

| SNP | Genotype | Controls | | | | Cases | | | |
| --- | --- | --- | --- | --- | --- | --- | --- | --- | --- |
|  |  | Actual | Expected | χ2 | P | Actual | Expected | χ2 | P |
| rs3790435 | TT | 5 | 2.19 | 5.00 | 0.08 | 15 | 12.20 | 1.09 | 0.579 |
|  | TC | 19 | 24.62 |  |  | 75 | 80.61 |  |  |
|  | CC | 72 | 69.19 |  |  | 136 | 133.20 |  |  |
| rs3790431 | AA | 87 | 87.26 | 2.38 | 0.305 | 204 | 203.59 | 0.33 | 0.849 |
|  | AG | 8 | 9.48 |  |  | 21 | 21.83 |  |  |
|  | GG | 1 | 0.26 |  |  | 1 | 0.59 |  |  |
| rs13306519 | CC | 66 | 65.01 | 0.48 | 0.786 | 149 | 149.81 | 0.13 | 0.939 |
|  | CG | 26 | 27.98 |  |  | 70 | 68.39 |  |  |
|  | GG | 4 | 3.01 |  |  | 7 | 7.81 |  |  |
| rs3206172 | CC | 78 | 77.94 | 0.00 | 0.998 | 186 | 186.86 | 0.48 | 0.786 |
|  | CG | 17 | 17.12 |  |  | 39 | 37.28 |  |  |
|  | GG | 1 | 0.94 |  |  | 1 | 1.86 |  |  |

**Supplementary Table 4** Genotype and allele frequencies for *LEPR* genes in obstructive sleep apnea (OSA) and control subjects

| SNP | Genotype | OSA group (N/%) | Controls (N/%) | χ2 | P |
| --- | --- | --- | --- | --- | --- |
| rs3790435 | TT | 15 (6.64%) | 5 (5.21%) | 6.65 | 0.036* |
|  | TC | 75(33.19%) | 19 (19.79%) |  |  |
|  | CC | 136 (60.18%) | 72 (75.00%) |  |  |
|  | T | 105(23.23%) | 29(15.10%) | 5.40 | 0.020* |
|  | C | 347(76.77%) | 163(84.90%) |  |  |
| rs3790431 | AA | 204 (90.27%) | 87(90.63%) | 0.46 | 0.838 |
|  | AG | 21 (9.29%) | 8 (8.33%) |  |  |
|  | GG | 1 (0.44%) | 1 (1.04%) |  |  |
|  | A | 429(94.91%) | 182(94.79%) | 0.004 | 0.950 |
|  | G | 23(5.09%) | 10(5.21%) |  |  |
| rs13306519 | CC | 149(65.93%) | 66(68.75%) | 0.65 | 0.695 |
|  | CG | 70(30.97%) | 26(27.08%) |  |  |
|  | GG | 7(3.10%) | 4(4.17%) |  |  |
|  | C | 368(81.42%) | 158(82.29%) | 0.069 | 0.793 |
|  | G | 84(18.58%) | 34(17.71%) |  |  |
| rs3206172 | CC | 186 (82.30%) | 78 (81.25%) | 1.13 | 0.712 |
|  | CG | 39 (17.26%) | 17 (17.71%) |  |  |
|  | GG | 1 (0.44%) | 1 (1.04%) |  |  |
|  | C | 411(90.93%) | 173(90.10%) | 0.109 | 0.742 |
|  | G | 41(9.07%) | 19(9.90%) |  |  |
